# Nutritional assessment and dietary intervention among survivors of childhood cancer: current landscape and a look to the future

**DOI:** 10.3389/fnut.2023.1343104

**Published:** 2024-01-31

**Authors:** Talia Feit, Elizabeth Beals, Smita Dandekar, Nina Kadan-Lottick, Lenat Joffe

**Affiliations:** ^1^Division of Pediatric Hematology/Oncology and Stem Cell Transplantation, Cohen Children’s Medical Center, Northwell Health, New Hyde Park, NY, United States; ^2^Department of Pediatrics, Cohen Children’s Medical Center, Northwell Health, New Hyde Park, NY, United States; ^3^Division of Pediatric Hematology/Oncology, Milton S. Hershey Medical Center, Penn State Health Children’s Hospital, Hershey, PA, United States; ^4^Departments of Oncology and Cancer Prevention and Control, Lombardi Comprehensive Cancer Center, Georgetown University, Washington, DC, United States; ^5^Department of Pediatrics, Donald and Barbara Zucker School of Medicine at Hofstra/Northwell, Hempstead, NY, United States

**Keywords:** childhood cancer, survivorship, health outcomes, nutritional status, nutritional assessment, dietary intake, dietary intervention

## Abstract

Over 85% of childhood cancer patients become long-term survivors. Still, cancer and its therapies are associated with a myriad of long-term complications such that childhood cancer survivors (CCS) endure excess disease burden, morbidity, and mortality throughout their lifetimes. Existing literature suggests that CCS maintain poor dietary intake and nutritional status. Thus, as childhood cancer cure rates continue to improve, the role of diet and nutrition in mitigating many of the most common adverse long-term health outcomes among CCS has gained significant interest. Herein we present an in-depth review of existing scientific literature evaluating dietary intake and nutrition status among CCS and its impact on treatment-related health complications; as well as contemporary intervention strategies aimed at overcoming distinctive barriers and improving deleterious lifestyle behaviors in this heterogeneous, at-risk population. Patient-specific, clinical, and systemic factors act as barriers to the timely conduct of comprehensive dietary/nutritional assessments and provision of tailored, risk-based recommendations. This Mini Review discusses the current state of the science, persisting research gaps, and opportunities for advancement of assessment and intervention strategies to address the unique needs of CCS.

Search Strategy: We searched PubMed for peer-reviewed articles with the search terms “pediatric cancer,” “pediatric malignancy,” “pediatric oncology,” “childhood cancer,” “survivorship,” “cancer late effects,” “long-term follow-up,” “body mass index,” “nutritional status,” “malnutrition,” “body weight,” “body weight changes,” “body composition,” “obesity,” “overweight “, “Mediterranean diet,” “DASH diet,” “processed foods,” “micronutrients,” “antioxidants,” “vitamin D,” “calcium,” “selenium,” “zinc,” “metabolic syndrome,” “heart disease,” “cardiovascular disease,” “cardiometabolic disease,” “hypertension,” “hyperlipidemia,” “HDL,” “LDL,” and “small dense LDL” from January 1, 1995, to July 21, 2023. We also selected relevant articles from our personal files and from reference lists of identified papers. We prioritized publications after 2013; however, commonly cited and highly regarded (defined by high citation count and journal impact factor) older publications were also included. Randomized controlled trials, observational studies, retrospective studies, meta-analysis, editorials, and review articles were included, whereas conference abstracts and case reports were excluded. We only searched for articles published in English, or those translated into English.

## Introduction

Although 5-year childhood cancer survival rates in the United States currently exceed 85% (1), curative treatments are associated with a myriad of physical and psychosocial late effects that can develop months to decades post-therapy. Childhood cancer survivors (CCS) have an excess burden of chronic health conditions, morbidity, and mortality compared with the general population (2, 3). Recently, Dixon et al. demonstrated that maintaining a healthy lifestyle, including a normal body mass index (BMI), is associated with reduced mortality risk among survivors (3). The long-term impact of nutritional status and dietary intake on health outcomes during and after treatment is gaining interest as a modifiable risk factor among CCS.

Anthropometric measures, such as BMI, are often used as a proxy for nutritional status. However, it is well-recognized that these are inadequate indicators of nutritional deficiency, particularly within the cancer population (4). Uniform and accessible nutrition assessment tools and intervention strategies for the clinical setting are lacking. Thus, nutritionally deficient patients are likely to go underrecognized and undertreated (4–7).

Evidence-based guidelines, including those published by the Children’s Oncology Group and the International Guideline Harmonization Group, have been established to guide risk-based late effects surveillance for CCS (8, 9). While these address modifiable lifestyle factors as they relate to specific late effects, they do not detail dietary guidelines directed at this population. Similarly, groups like the American Cancer Society provide generalized recommendations, but do not address CCS-specific challenges (7). Lifestyle interventions are often difficult to implement (10). Establishing cohesive CCS-directed dietary guidelines is essential to providing appropriate and effective intervention for this vulnerable group (11).

This Mini Review presents a timely, expansive discussion on the role of nutrition and diet in childhood cancer survivorship, barriers for effective intervention among CCS, and evolving implementation and dissemination strategies aimed at this population. Promoting scientific understanding and enhancing clinician knowledge in this this area is a critical component of safeguarding long-term health and quality of life for this at-risk population.

## Modifiable lifestyle factors and chronic health conditions among CCS

Exposure to antineoplastic therapies including conventional chemotherapies, targeted agents, radiation therapy, and surgery can result in long-lasting health complications (2). Bhakta et al. demonstrated that by age 45 CCS have twice the chronic disease burden compared to the general population. On average, survivors experience 4–5 severe/disabling, life-threatening, or fatal chronic health conditions by age 50 (2). Cardiovascular disease (CVD) serves as the largest contributor to noncancer-related premature mortality (3, 12). CCS are also more likely than peers to suffer from one or more CVD risk factors, including hypertension, dyslipidemia, diabetes, and metabolic syndrome (12–15). Chow et al. found that CVD risk factors are underdiagnosed in >25% of adult CCS and undertreated ~20% of the time, with the greatest risk associated with elevated BMI and/or adverse lifestyle factors (12). Although certain therapeutic exposures can predispose survivors to high BMI, overweight/obesity rates among CCS are similar to those of the general population (16–19). Large national and international population-based cohort studies have contributed tremendously to our understanding of late effects associated with individual therapies (20–23). These have informed the creation of consensus-based guidelines intended to mitigate the development of chronic health conditions in the survivor population (8, 9). Dietary habits have the potential to significantly alter the trajectory for cardiovascular health and metabolic disease among CCS (13). A better understanding of existing CCS dietary patterns is essential to informing the development of directed guidelines and improving survivor health outcomes (6, 18, 24).

## Dietary patterns and interventions among CCS

The majority of existing observational and interventional nutrition-focused studies among CCS were conducted in resource-rich countries (25–29). In the United States, poor dietary guideline adherence rates among CCS are similar to those of the general population (30). However, given survivors’ excess risk for chronic health conditions, poor diet carries greater health consequence (13). In a small, single-institution study Zhang et al. utilized a 24-hour diet recall tool and found that CCS, particularly those >10 years from diagnosis, exhibit poor diet quality, including insufficient fruit, vegetable, and dietary fiber intake (11). Survivors consume excess carbohydrates and fats, while maintaining an insufficient intake of protein, fiber, calcium, vitamin D, folic acid, and vitamin B12 (11, 30, 31). In a cross-sectional study of Swiss CCS, Belle et al. found that, irrespective of individual CVD risk, daily sodium intake was nearly double, while potassium intake was consistently below, recommended daily values (32). Notably, among ~200 acute lymphoblastic leukemia (ALL) CCS evaluated with food frequency questionnaires in the PETALE Study, ultra-processed foods [NOVA classification(33)] made up 50% of survivors’ dietary intake (34–36).

Consumption of ultra-processed foods such as soft drinks, packaged snack foods, flavored yogurts, and commercial breads and cereal has been associated with increased risk of CVD within the general population (34, 37, 38). Moreover, abnormal lipid marker levels [i.e., elevated low-density lipoprotein (LDL), small dense LDL and insufficient high-density lipoprotein (HDL)] are directly linked with atherosclerosis and worse cardiometabolic health (39–41). PETALE Study CCS with higher ultra-processed food intake were more likely to have elevated triglyceride and inflammatory marker levels (34). Additionally, higher fast-food and calorie intake were associated with lower HDL levels, while higher protein, red/white meat, and fruit consumption decreased the odds of low HDL in this population (36). ALLIFE Study authors further demonstrated that higher small dense LDL is associated with significantly lower HDL, as well as increased triglycerides, visceral obesity, insulin resistance, and metabolic syndrome among ALL CCS (42). The Healthy Eating Index (HEI; higher scores indicative of better adherence to the Dietary Guidelines for Americans) has been linked with improved BMI values and reduced CVD risk in the general population (43, 44). Similarly, Lan et al. found that higher HEI scores mitigate CVD risk among adult male CCS (45).

### Vitamins, minerals, and antioxidants

PETALE Study investigators determined that CCS with a higher estimated intake of the minerals zinc, copper, and selenium, as well as the B vitamins niacin and riboflavin, were less likely to have low HDL (36). Selenium and zinc are trace elements that serve as enzymatic cofactors and their suggested cardioprotective effect may relate to their role in reducing mitochondrial dysfunction (4, 46). In a systematic review examining the role of antioxidant nutrients in CVD post-cardiotoxic chemotherapy, Zhang et al. reported that polyphenols such as resveratrol and curcumin may help reduce free radical-related damage and consequent cardiac dysfunction (4, 47). Vitamin D and calcium intake have also been suggested to impact long-term survivor health outcomes (42, 48, 49). Serum vitamin D levels were inadequate among both adult and pediatric PETALE Study participants (50). Among females, serum vitamin D level was positively associated with HDL concentration, but was not associated with other cardiovascular risk factors, such as metabolic syndrome, insulin resistance, glycemia, and triglyceride levels (49). Existing studies suggest that certain micronutrients may be associated with cardiovascular health in the survivor population, but data are limited. Additional research is needed to elucidate pathophysiologic mechanisms driving the relationships between certain nutritional components and clinical outcomes, and to confirm these findings in larger survivor populations.

### Dietary interventions

Specific dietary interventions investigated among CCS are similar to those studied within the general public (51–56). Several randomized controlled trials among high-risk adults have demonstrated that Mediterranean diet adherence reduces the risk of myocardial infarction, stroke, and CVD-associated death in the general population (51, 57). The Mediterranean diet is characterized as being calorie-unrestricted, rich in fish, dairy, and plant-derived foods such as whole grains, vegetables, fruits, legumes, and olive oil, and limited in meat consumption (58). In a cross-sectional study of 118 ALL CCS, Tonorezos et al. found that greater adherence to the Mediterranean diet was associated with significantly lower visceral and subcutaneous adiposity, smaller waist circumference, and lower BMI (52). Moreover, the odds of developing metabolic syndrome fell by 31% for every one-point increase in a Mediterranean diet adherence score (52). Similarly, in a SJLIFE cohort study, Lan et al. demonstrated that adult CCS experienced an 8% CVD risk reduction for every one-point score increase in an alternate Mediterranean diet (aMED) score (45). Collectively, these findings indicate that the role of diet and nutrition in mitigating CVD risk in the survivor population is likely multifactorial, relating both to diet quality and healthy weight maintenance/body composition. Further research is needed to identify optimal dietary and nutritional recommendations addressing the unique needs of CCS.

## Risk factors for poor lifestyle habits

**Figure 1:**
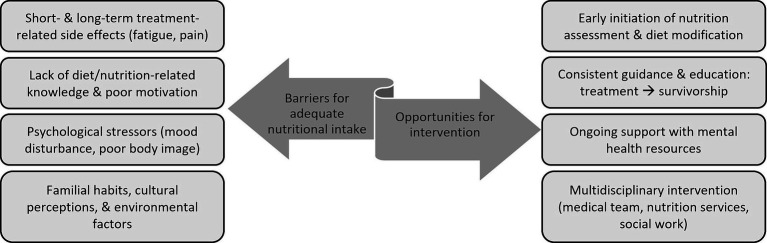
Patient-specific barriers and opportunities for nutritional intervention (59–63).

Understanding the barriers precluding CCS from adhering to healthy lifestyle recommendations is vital to establishing meaningful intervention strategies (Figure 1) (64, 65). Mizrahi et al. found that only 25% of pediatric CCS achieve recommended physical activity levels, citing fatigue, lack of motivation/time, uncertainty, and pain as major limitations (66). Johnson et al. further demonstrated that among brain tumor survivors high BMI can exacerbate fatigue and stress (59). Literature indicates that, consequent to treatment side effects, CCS are likely to remain choosy/selective eaters, which further promotes detrimental habits and diet quality (64). Establishing cancer treatment risk-based nutrition recommendations and ensuring adequate patient engagement will enable the provision of meaningful patient education that has potential to positively impact individual health perceptions and long-term lifestyle behaviors (65).

Adolescent and young adult (AYA) CCS present a unique challenge to incorporating healthy behaviors after treatment. Rates of physical activity, smoking, and illicit drug use among AYA survivors are comparable to those of general population peers (60). However, because survivors’ risk for cardiometabolic disease is much greater, engaging in risky behaviors is more likely to result in poorer health outcomes (60). Physical, mental, and emotional health sequelae of cancer care can result in a long-lasting negative impact on survivors’ body image, and subsequently their lifestyle habits (61). Psychological support services are critical to helping survivors navigate the mental and emotional challenges that often accompany cancer treatment. Limited access to these services serves as another barrier to proper adherence to healthy lifestyle choices (62, 65).

## Current intervention recommendations and strategies

Validated and accessible interventions focusing on CCS dietary habits are lacking. Significant demographic and medical heterogeneity within the survivor population, lack of consensus regarding optimal timing for intervention, paucity of uniform assessment tools / scalable interventions, and logistics surrounding long-term follow-up programs are among the obstacles to creating uniform CCS-directed nutritional assessments and recommendations (Table 1) (7, 16, 63, 65). Properly addressing each of these barriers will enable the delivery of critical nutritional assessment, education, and intervention to successfully modify adverse health behaviors before they become long-term practices. Existing literature indicates that elevated BMI and poor lifestyle choices during treatment, including diet, exercise, and sleep, are likely to persist in the post-treatment phase and negatively impact long-term survivor health (10, 16, 24, 74). Hence, many advocate that lifestyle interventions should be incorporated quickly after cancer diagnosis (24, 67). Yet, historically, most dietary intervention studies among children with ALL, a population with known predisposition for obesity and CVD, have not been introduced prior to the maintenance phase of treatment (75). More contemporary investigations, however, are demonstrating that dietary intervention among newly diagnosed pediatric oncology patients is not only feasible, but also confers a positive impact (67). Collectively, these findings highlight the importance of consistent health behavior education beginning at diagnosis and persisting far beyond completion of treatment.

**Table 1:** Key components of diet intervention strategies(67–73).

Component	Subtype	Benefits
Target population	Survivors only	Enables comprehensive focus on survivor-specific attributes (age, sex) & stressors (motivation, uncertainty, time)
	Survivors & caregivers	Addresses family dynamics & perceptions Considers environmental & resource barriers
	Individual survivor populations	Recommendations tailored to cancer / treatment-specific late effects
	Mixed survivor populations	Permits dissemination & implementation among broader populations
Timing of implementation	Diagnosis / Initiation of treatment	Promotes sustained habits & long-term success
	After completion of therapy	Survivors & caregivers may have greater ability to focus on long-term health needs
Assessment & intervention mechanisms	In-person	Enables use of objective assessment measures Facilitates patient/provider communication & connection
	Remote	Cost-effective Convenient & efficient Facilitates wide-reaching & frequent follow-up

CCS are a highly diverse population of pediatric and adult patients with differing familial, cultural, socioeconomic, and geographic backgrounds. A variety of methods aiming to better understand overarching survivor needs, as well as identify optimal communication and education delivery strategies, have been investigated (10, 24, 64). In a qualitative study, Clarke et al. noted that survivors desire general, as well as cancer-directed, nutrition education and guidance (63). The authors suggest using either an in-person or remote multi-step approach, with an initial focus to evaluate adherence to, and benefit from, general dietary guidelines. Subsequently, recommendations would be tailored to the individual needs of the patient (63). Data regarding the optimal modality for delivering effective and sustainable dietary counseling and intervention remain limited. Self-Management Education programs (SMEPs), which can be delivered as in-person sessions (individualized or group-based) or web-based modules are designed to enhance personal health knowledge, self-efficacy, and confidence among those contending with chronic health conditions (62). It has been suggested that CCS, and AYAs in particular, could significantly benefit from such technology-based programs (62). In fact, in recent years CCS studies have increasingly opted for remote (telephone, mobile application, telehealth) intervention strategies as these are typically more cost-effective, accessible to those living in rural and/or remote areas, and preferred among younger groups (60, 68–71). Nevertheless, Touyz et al. found that both adolescents and their parents preferred face-to-face intervention to a web-based one (68). The lack of consensus regarding an optimal intervention approach is compounded by studies like that of Alchin et al., who found that AYA survivors exhibit limited recall and poor adherence to healthcare recommendations despite the use of a variety of communication modalities for education delivery (70). In an effort to overcome some of these unique hurdles contemporary investigations are incorporating novel approaches alongside more conventional strategies, such as creating a CCS-directed cookbook and providing guided lifestyle coaching sessions (71, 76).

The physical and emotional toll of childhood cancer impacts not only patients, but also caregivers and families alike. As such, parental approach often changes in the course of cancer care and caregivers tend to become more permissive with respect to their sick child’s eating habits (63, 64). Provision of timely caregiver education underscoring the potential health impact associated with such decisions is critical (69). Thus, lifestyle interventions must be designed to not only consider the complex needs of patients, but also caregiver perception and existing family practices. In a single-institution study Cohen et al. demonstrated that compared to parents of matched population peers, parents of pediatric CCS are more likely to struggle with providing proper dietary intake (72). CCS were reported to consume less fruits and vegetables, more junk food, and bigger portion sizes compared to their peers (72). Pilot studies focusing on diet/nutrition education and intervention among parents of survivors have yielded encouraging results, demonstrating feasibility and preliminary signs of efficacy in remediating poor dietary habits among CCS and families (73, 76, 77). Though larger randomized clinical trials are needed, the successful implementation and initial findings from these, primarily remote, parent-led initiatives demonstrates their role in modifying patient-specific as well as familial dietary patterns (73, 76, 77). Additionally, these reinforce the utility of structured web-based curriculums, like the HEAL initiative, in providing wide-reaching, dedicated, and cost-effective lifestyle guidance and education to parents of CCS (69).

The availability and scope of long-term survivorship care after childhood cancer is highly variable and depends on factors such as geographic location, treating institution, and local/regional resources (62, 70). Infrequent clinic visits, limited time, and insufficient resources are several systemic factors that limit healthcare providers’ ability to provide ongoing, comprehensive education/counseling and meaningful lifestyle interventions (63). A necessary component of addressing these hurdles is the identification of dietary intake and nutrition quality assessment tools that enable efficient evaluation in the clinical setting. Current assessment mechanisms include both patient-reported (food frequency questionnaires, diet recall) and clinically collected (urine spot collection, anthropometric measures, body composition assessment) measures (10, 17, 32, 78, 79). While patient-reported assessments are more cost-effective and easier to implement across varying study settings, they are also more prone to bias than objectively assessed data (32). It is likely that a combination of modalities is ultimately needed to best assess dietary intake and nutritional status among CCS. First, however, further investigation is needed to better elucidate the utility of each of these methodologies in the CCS population.

## Discussion

CCS are a diverse and medically-complex group for whom modifiable lifestyle factors, including nutritional intake, have the potential to improve long-term health and quality of life (72). Large-scale investigations conducting detailed nutritional assessments among heterogeneous groups of CCS are limited. However, existing literature indicates that significant portions of both pediatric and adult CCS maintain poor dietary habits, including an excess consumption of ultra-processed foods and insufficient intake of essential micro- and macronutrients. This then augments the risk of excess weight gain, hypertension, hyperlipidemia, diabetes, and CVD in this population (34, 36). Identifying dietary patterns that minimize adverse health outcomes among CCS is an actively evolving area of research, with observational studies presently serving as the primary source of data. Research suggests that minimally processed, varied diets rich in whole grains, fruits, vegetables, and lean sources of protein are likely beneficial for most CCS (52). While such diets have been shown to mitigate chronic health conditions within the general population, randomized clinical trials are needed to evaluate the relationship between specific dietary recommendations and survivor health outcomes. Moreover, this narrative review underscores the existing gap in large-scale investigations essential to the development of uniform, evidence-based nutrition assessment strategies, education/counseling approaches, and risk-based dietary recommendations directed at the unique needs of CCS.

In addition to a lack of standardized dietary guidelines for CCS, there are also systemic, environmental, and patient-specific barriers hindering successful and reproducible implementation of nutrition-based interventions in this population. These include poor healthcare access, limited resource availability, infrequent and/or ineffective provider communication, lack of education, diverse familial/cultural perceptions, physical health limitations, and psychological burden (74, 80). Investigators have identified several key strategies to address these issues. Of primary importance is the early initiation of nutrition-focused education among both patients and caregivers. Promotion of national guideline recommended dietary habits during the early phases of cancer care optimizes the likelihood CCS will adhere to these long-term, and mitigates the challenges associated with reversing long-standing unhealthy behaviors (10, 67). Incorporating continued dietary/nutrition screening and counseling, whether in-person or remotely, into survivorship clinical care is also paramount to ensuring sustained compliance with healthy eating habits (63, 68). Further intervention strategies are needed to better address individual health status, familial perceptions, and coexisting psychosocial stressors that may be impacting adverse lifestyle choices. Clinicians should apply sensitive communication skills to convey long-term health risks, and utilize available local, regional, and national resources to guide and support patients and families through lifestyle modification (70).

CCS are a growing population with excess chronic disease burden, morbidity, and mortality associated with their cancer treatment. Establishing survivor-directed lifestyle screening recommendations and interventions is critical to improving long-term health outcomes for this at-risk population. The central role of lifestyle factors, including nutritional status and diet quality, in shaping CCS health is an area of burgeoning scientific interest and inquiry. Extensive future research is needed to bridge existing preclinical, clinical, and systemic knowledge gaps. These investigations will facilitate the construct of cohesive CCS lifestyle recommendations, and thereby enhance medical care and well-being in this population.

## Author contributions

TF: Writing – original draft, Conceptualization, Investigation, Writing – review & editing. EB: Writing – original draft, Writing – review & editing, Investigation. SD: Writing – review & editing. NK-L: Writing – review & editing. LJ: Writing – original draft, Conceptualization, Investigation, Supervision, Writing – review & editing.
